# A guide to improve your causal inferences from observational data

**DOI:** 10.1177/1474515120957241

**Published:** 2020-10-10

**Authors:** Koen Raymaekers, Koen Luyckx, Philip Moons

**Affiliations:** 1 School Psychology and Development in Context, KU Leuven, Belgium; 2 Research Foundation Flanders (FWO), Belgium; 3 UNIBS, University of the Free State, South Africa; 4 Department of Public Health and Primary Care, KU Leuven, Belgium; 5 Institute of Health and Care Sciences, University of Gothenburg, Sweden; 6 Department of Paediatrics and Child Health, University of Cape Town, South Africa

**Keywords:** Research methods, causality, repeated measures, nursing research, quantitative

## Abstract

True causality is impossible to capture with observational studies. Nevertheless, within the boundaries of observational studies, researchers can follow three steps to answer causal questions in the most optimal way possible. Researchers must: (a) repeatedly assess the same constructs over time in a specific sample; (b) consider the temporal sequence of effects between constructs; and (c) use an analytical strategy that distinguishes within from between-person effects. In this context, it is demonstrated how the random intercepts cross-lagged panel model can be a useful statistical technique. A real-life example of the relationship between loneliness and quality of life in adolescents with congenital heart disease is provided to show how the model can be practically implemented.

## Learning objectives

- Understanding the value of repeated measures, investigating the temporal sequence of effects and distinguishing within from between-person effects in observational data;
- Understanding how the random intercepts cross-lagged panel model (RI-CLPM) can be a useful statistical technique to tackle within-person research questions;
- Understanding when it is appropriate to implement findings from the RI-CLPM in clinical practice.

## The problem: tackling causal questions in observational studies

Health researchers and practitioners are ultimately interested in unraveling the key factors that drive change in patients’ health status, behaviour and general wellbeing. Being able to identify and manipulate such causal factors is fundamental for designing effective clinical interventions. Variable A can be considered a causal factor to variable B when the conditions of covariation, temporal sequence and the confounding variable problem are met (Table 1).^1^ Although randomised controlled trials are considered the benchmark designs for inferring causality, they too have their methodological flaws and are not always feasible for both practical and ethical reasons.^2,3^

**Table 1.** Variable A has a causal effect on variable B when the following three conditions are met.^1^

Covariation	When variable A changes, variable B changes as well
Temporal sequence	Change in variable A occurs earlier in time than change in variable B
Confounding variable problem	The relationship between A and B is not the result of a causal factor they both share

A worthy alternative is the longitudinal observational study design, in which patients are observed repeatedly over time, for example by means of self-administered questionnaires. Such data in which a set of variables is measured repeatedly are referred to as repeated measures data. Repeated measures data allow us to investigate whether the causality conditions on covariation and temporal sequence (points 1 and 2 in Table 1) among two variables are met. Furthermore, with such data it is possible partially to mitigate the effects of potential confounding variables (point 3 in Table 1). Hence, although repeated measures data do not allow investigating true causality, they can be a valuable option for researchers who want to tackle causal questions.^3,4^ However, this asset comes with a cost. Repeated measures data are complex and researchers do not always have the knowledge, skill and software support to use such data to their full potential.

A first complexity that arises is how to take the temporal sequence of effects into account. To do so, one must control for the stability across time of the dependent variables. Moreover, many relationships among variables in the field of cardiovascular nursing (and other fields) are in fact bi-directional. For example, psychological wellbeing has been found to reduce the risk of developing heart disease.^5^ Heart disease, in turn, has been found to impact psychological wellbeing negatively.^6^ Basic statistical techniques, such as linear regression and analysis of variance (ANOVA), only assess a single direction of effects, being from the independent to the dependent variable. Therefore, they cannot test the possibility of both variables predicting each other. Furthermore, when mutual influences among variables exist, it can be of interest to compare the strength of both directions of effect directly to see which variable is the main driving force in this bi-directional relationship.^7^

The second and likely most confusing complexity surrounding repeated measures data arises from its two levels of analysis, being the between and within-person levels. At the between-person level of analysis it is possible to investigate research questions that focus on differences between persons. An example of such a question is: do individuals who experienced a cardiac event have poorer sexual functioning compared to individuals who did not experience such an event?^8^ At the within-person level of analysis it is possible to investigate research questions that focus on changes within persons. For example, when someone experiences a cardiovascular event, does that impact his/her sexual activity?^8^ Here, the main interest is on the within-person relationship among these variables, that is, whether a cardiac event has a negative impact on sexual functioning.^9^

Distinguishing effects at the within and between-person level is important given that these are two different things. Mixing up these two levels of inference may lead to erroneous conclusions concerning relationships among variables of interest. One example that demonstrates this issue is the relation between exercising and cardiac arrests.^9^ People who exercise more have a lower risk of cardiac arrests than people who exercise less (i.e. a between-person effect). At the same time, however, a cardiac arrest is more likely to happen on days in which an individual is exercising more than he/she is used to than on days in which an individual is exercising less (i.e. a within-person effect). In this example the valence of the relationship at the within-person level is opposite to that at the between-person level. However, in many real-life examples, it is also possible that relationships at both levels are in the same direction but differ in strength, or do not differ at all. For example, Masselink et al.^10^ found that adolescents with lower self-esteem at baseline had more depressive symptoms later in time, compared to adolescents with more self-esteem at baseline (i.e. a between-person effect). Also, when adolescents experienced lower self-esteem than they usually would, they experienced more depressive symptoms than they usually would later in time (i.e. a within-person effect).

In sum, to make stronger claims about within-person effects in longitudinal observational studies, researchers must: (a) repeatedly assess the same construct within persons to get repeated measures data; (b) consider the temporal sequence of effects; and (c) use an analytical strategy that distinguishes within from between-person effects. Many studies with longitudinal observational designs do not fulfill all three conditions and thus are limited to discussions of association and not causation. Recent examples from the *European Journal of Cardiovascular Nursing* include longitudinal studies lacking repeated measures,^11^ not considering the temporal sequence of effects,^12^ or not differentiating within and between-person effects.^13^

## A solution: the RI-CLPM

**Figure 1.**
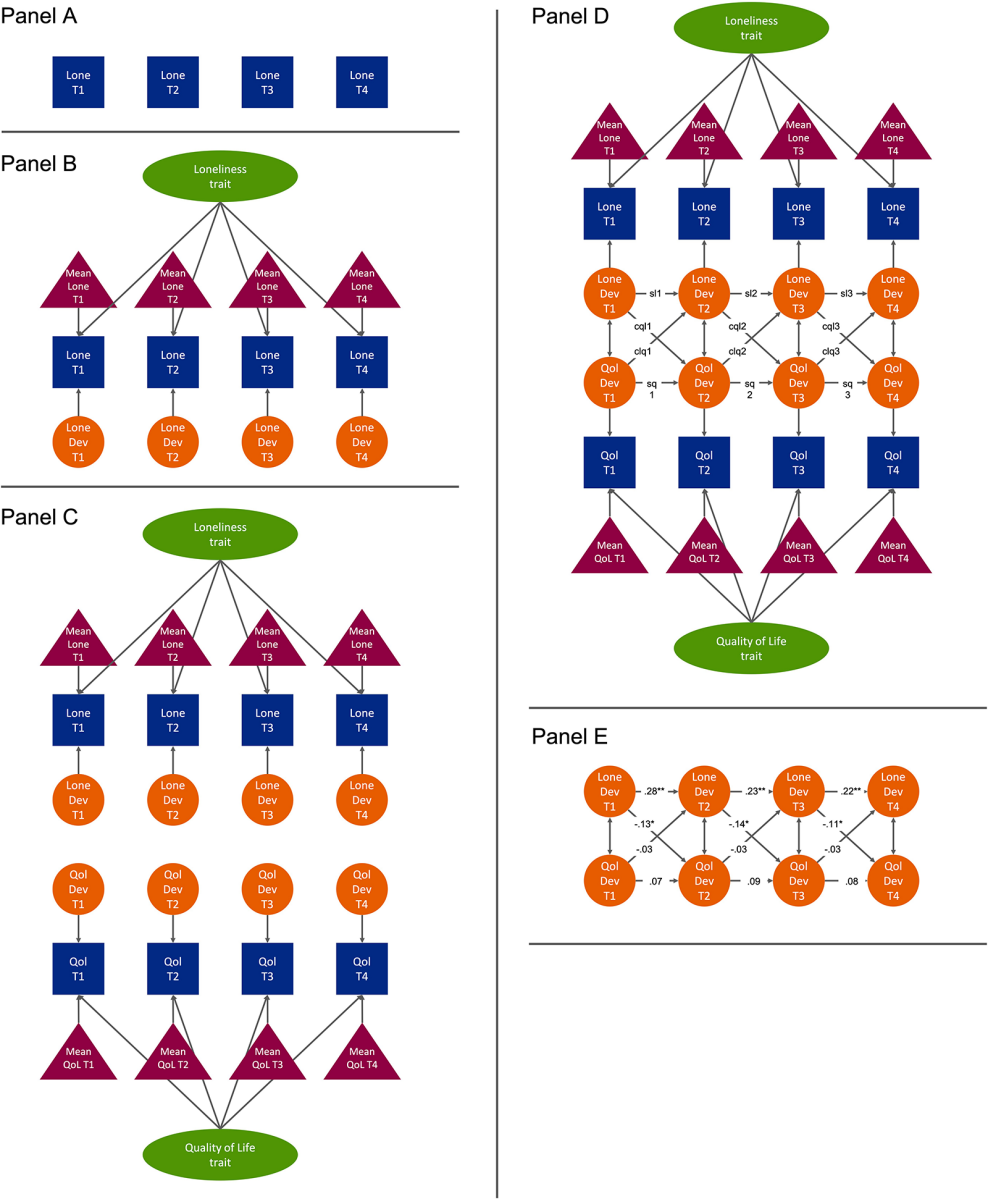
Example of a random intercepts cross-lagged panel model with two variables (loneliness and quality of life) and four time points. Observed and latent variables are depicted by squares and circles, respectively. The group means are depicted by triangles. The covariances are depicted by double-sided arrows. For reasons of clarity, the residuals are not displayed.

Within the structural equation modelling (SEM) branch of statistics, several models exist that can incorporate temporal sequence and differentiate within and between-person effects (see Hamaker et al.,^7^ Mund and Nestler,^14^ Berry and Willoughby^15^ or Orth et al.^16^ for an overview). The classic cross-lagged panel model (CLPM) takes into account covariation and the temporal sequence of variables but does not differentiate within and between-person levels of analysis. In the present paper, the focus is on the RI-CLPM, which is a direct extension of the classic CLPM.^7^ Figure 1 depicts a representation of the RI-CLPM. In the following section, the model will be explained step by step.

Imagine four repeated measures of loneliness in a group of patients. The four repeated measures of loneliness are represented in Figure 1, panel A by ‘lone T1’, ‘lone T2’, ‘lone T3’ and ‘lone T4’. The RI-CLPM breaks down each of these observed measures into three new latent (i.e. not directly measured) variables (Figure 1, panel B). These variables are: (a) The temporal group mean of loneliness at each time point (represented by a red triangle). As this is a temporal group mean, its value stays constant across persons and differs across time points. (b) A person-specific loneliness trait that is considered time-invariant (‘loneliness trait’, green oval). (c) A person and time-specific deviation from that trait (e.g. ‘lone dev T1’, orange circles). In other words, the RI-CLPM predicts that someone’s observed loneliness score (blue squares) at time 1 is the sum of the group mean of loneliness (red triangle) at time 1, the person-specific loneliness trait score (green oval) and the person-specific deviation from that trait at time 1 (orange circle). Thus, the fact that some individuals are lonelier than others (i.e. the between-person effect of loneliness) is captured by variable ‘loneliness trait’. The fact that an individual may feel lonelier at one point in time than at another time point (i.e. the within-person effect) is captured by variables ‘lone dev T1’, ‘lone dev T2’, ‘lone dev T3’ and ‘lone dev T4’. The model can be expanded with other variables of interest, such as quality of life (QoL) (Figure 1, panel C).

The part of CLPMs that is of most substantive interest are the cross-lagged paths, being the relationship between two variables (e.g. loneliness and QoL) over time (Figure 1, panel D). In the RI-CLPM, the cross-lagged effects are modeled among the within-person deviations, rather than among the observed variables themselves, as the observed variables represent an amalgam of within and between-person variance that can be difficult to interpret. One such path is ‘cql1’, with ‘c’ standing for ‘cross-lagged path’, ‘ql’ standing for ‘QoL regressed on loneliness’ (i.e. the path from loneliness to QoL) and ‘1’ because time-wise it is the first of three cross-lagged paths from loneliness to QoL.

Thus, the cross-lagged effect ‘cql1’ denotes the within-person relationship between their loneliness at time point 1 (T1) and their QoL at time point 2 (T2). One may also note that ‘QoL dev T2’ is not only predicted by ‘lone dev T1’ but also by itself at a previous time point, i.e. ‘QoL dev T1’. This is to control for previous values of QoL, the so-called ‘stability effect’, captured by ‘sq1’. When this stability effect would be high (i.e. close to 1), this would mean that individuals’ QoL would only slightly deviate from their QoL trait over time.

For researchers who want to implement the RI-CLPM in their own research, there are some additional points to bear in mind. These points are detailed in Supplementary File 1.

## Software

A good software choice to implement the RI-CLPM is the ‘Lavaan’ package in R.^17^ It is freely available, and there already exist some example syntaxes in the scientific community that may be helpful when practically implementing the model (e.g. Mund and Nestler).^14^ Information and tutorials on how to use Lavaan can be found here: http://lavaan.ugent.be/. Another software option with some example syntax available is Mplus.^18^

## Example of the RI-CLPM

To demonstrate the use of the RI-CLPM, directionality of effects between loneliness and QoL were investigated in adolescents with congenital heart disease (CHD). The present example builds on a paper by Luyckx et al.,^19^ in which (among other variables) the directionality of effects between loneliness and QoL across three data waves were investigated using classic CLPM. Four repeated measures of QoL and loneliness were obtained separated by three 9-month time intervals. A total of 429 cases were available for analysis at time (T)1, 398 at T2, 366 at T3, and 337 at T4.

The means, standard deviations, within and between-time correlations of loneliness and QoL are provided in Table 2. Providing means and correlations (and/or variances and covariances) is good practice, as this information allows other researchers to replicate the analyses and obtained results from the RI-CLPM. Before estimating the actual model, researchers may want to decide on how to handle missing data and check some further assumptions. To handle missing data in the present example, the parameters were estimated using full information maximum likelihood (FIML). FIML produces asymptotically unbiased parameter estimates under the assumption that data are missing at random or missing completely at random. When data are not missing at random, the estimates obtained through FIML should be interpreted with caution. Missing not at random implies that the probability of having missing values for a variable depend on the missing values themselves.^20,21^ As both the loneliness and QoL variables are not normally distributed, maximum likelihood estimation with robust standard errors (MLR) was used to estimate the model. Both FIML and MLR estimation is straightforward to specify in the Lavaan syntax. The R-code that was used to specify the model is provided in Supplementary File 2.

**Table 2.** Means, standard deviations and correlations.

Variable	M	SD	1	2	3	4	5	6	7
1. Loneliness T1	1.78	0.64							
2. Loneliness T2	1.84	0.67	0.62**						
3. Loneliness T3	1.77	0.69	0.53**	0.60**					
4. Loneliness T4	1.83	0.68	0.56**	0.60**	0.68**				
5. Qol T1	82.64	11.89	−0.34**	−0.28**	−0.31**	−0.31**			
6. Qol T2	82.02	11.24	−0.32**	−0.32**	−0.28**	−0.34**	0.53**		
7. Qol T3	81.06	10.63	−0.26**	−0.34**	−0.49**	−0.42**	0.54**	0.52**	
8. Qol T4	81.46	11.28	−0.31**	−0.29**	−0.36**	−0.41**	0.48**	0.44**	0.59**

M: mean; SD: standard deviation; Qol: quality of life.

* *P*<0.05; **P<0.01.

All stability and cross-lagged paths between adjacent time points, and all within-time associations were estimated among the latent within-person variables (Figure 1, panel E). To make the model more parsimonious, and as there were no strong a priori theoretical expectations that the effects would be different across waves, all stability and cross-lagged paths were fixed to be equal across time (e.g. cql1=cql2=cql3=cql, and sl1=sl2=sl3=sl). This assumption could be formally tested by comparing a model in which these paths are fixed with a model in which these paths are estimated freely. If the model with fixed paths does not fit significantly worse than the freely estimated model, the fixed model would be preferred as it is the more parsimonious one. Supplementary File 1 provides more information on fixing SEM paths. The fit indices indicated excellent model fit (χ²(17)=15.84, *P*=0.535; root mean square error of approximation (RMSEA) 0, 90% confidence interval (CI) (0.44); standardized root mean square residual (SRMR) 0.034; comparative fit index (CFI)=1). Figure 1, panel E shows the standardised parameter estimates for the cross-lagged and stability effects. The standardised cross-lagged paths from loneliness to QoL were significantly different from zero, with *P*<0.05 and cql1 = −0.13, cql2 = −0.14 and cql3 = −0.11. Although unstandardised cross-lagged paths were fixed over time, the estimates of the standardised cross-lagged parameters differ slightly due to the standardisation process.

The results thus suggest that loneliness predicts worse QoL over time, rather than the other way around in adolescents with CHD. Although this didactical example was conducted using real-life data, future studies should replicate this effect in other samples as well before strong conclusions can be drawn on the within-person relationship between loneliness and QoL in adolescents with CHD.

## Reporting

First, it is important to report the fit indices of the model. If the model does not fit the data adequately, researchers must be especially cautious when interpreting the obtained parameter estimates. Kline,^22^ among other authors, covers basic principles of SEM and provides an overview of several fit indices and how to use and interpret them. Rules of thumb for acceptable model fit are the RMSEA <0.08, SRMR <0.10, CFI >0.90 and the normed χ² <2. The normed χ² is the model’s χ² value divided by its degrees of freedom. Second, as specified before, it is good practice to report the means, standard deviations, and correlations among the variables included in the model. Finally, one must at least provide the cross-lagged estimates, as these are of substantive interest when using the RI-CLPM. It is recommended also to report the stability estimates and the correlations among the latent trait variables.

## Conclusion

The purpose of the present paper was twofold. The first aim was to emphasise the importance of repeated measures, investigating the temporal sequence of effects, and distinguishing within from between-effects when working with longitudinal observational study designs. The second aim was to show how the RI-CLPM could be implemented to model the temporal sequence among variables and differentiate the different levels of analysis. In sum, the RI-CLPM can be a useful tool for cardiovascular nursing researchers who want to answer causal research questions.

## Supplemental Material

10.1177_1474515120957241_Supplementary_Material – Supplemental material for A guide to improve your causal inferences from observational data Click here for additional data file. Supplemental material, 10.1177_1474515120957241_Supplementary_Material for A guide to improve your causal inferences from observational data by Koen Raymaekers, Koen Luyckx and Philip Moons in European Journal of Cardiovascular Nursing
