# Supplementary material for: A guide to improve your causal inferences from observational data
Source: Eur J Cardiovasc Nurs. 2020 Oct 10;19(8):757–62. doi: 10.1177/1474515120957241 (PMC7817987; doi:10.1177/1474515120957241)
Supplement: 10.1177_1474515120957241_Supplementary_Material – Supplemental material for A guide to improve your causal inferences from observational data [file 10.1177_1474515120957241_Supplementary_Material.pdf]

**Supplemental material 1**

The following considerations concerning the RI-CLPM may serve as a guideline for researchers who want to implement the model in their own research. For a more detailed explanation of the model and its capabilities, the reader is referred to the manuscript by Hamaker, Kuiper [1]. First, the repeated measures are depicted in Figure 1 as manifest variables (i.e., in squares, directly observed). However, one could also specify a RI-CLPM with repeated measures of latent variables, that is, including a measurement part in the model. Second, modeling cross-lagged relations among more than two variables is possible as well. Third, control variables may be added by regressing the within-person factors on control variables of interest (e.g. age, sex, disease complexity), thereby providing a partial solution for the confounding variable problem. Fourth, many times, the cross-lagged and/or stability effects (among variables X and Y) are fixed over time (e.g. cyx1=cyx2=cyx3=cyx; sx1=sx2=sx3=sx). By doing this, the model becomes less complex and gains degrees of freedom, which is good. However, fixing paths to be equal across time must make sense in the realm of researchers' hypotheses, which is not always the case. When there are strong expectations that the relationship between two variables or the stability of a variable may change across the different time-intervals, fixing the model's paths across time may result in poor model fit. Fifth, the model can take into account unequally spaced time-intervals between observations, but more advanced SEM techniques that model time continuously may be more suited in such case[2]. Sixth, the model requires at least three waves of data to be identified, and even more waves are preferred to increase statistical power and allow for a more flexible specification of the model[3, 4]. Seventh, one's sample size needs to be large enough in order for the estimates to be reliable and to achieve large enough statistical power (this applies to SEM models in general). Ideally, one should conduct a power analysis to obtain an idea of the required sample size. An alternative, but less preferred, option is using a rule of thumb. One such rule states that the ratio of the sample size to the number of freely estimated parameters in the model should exceed five[5]. Finally, to practically implement the model, researchers must impose some restrictions on the variance structures of the included variables. For example, all measurement error variances of the observed variables need to be fixed to zero, since one assumption of the RI-CLPM is that all the observed variance can be split into a within- and a between-person variance component. Sometimes the estimation of parameters in SEM models (in general, thus not restricted to RI-CLPMs) may result in impossible values like negative variances. Such impossible values are also referred to as Heywood cases, for which several possible causes may exist[6]. For Heywood cases that do not significantly differ from zero, a simple solution is to fix their values to zero. Readers are referred to the R-code in Supplementary Material 2 for a practical example of how fixing parameters at a certain value can be done.

**References**

1. Hamaker EL, Kuiper RM and Grasman RP. A critique of the cross-lagged panel model. *Psychol Methods* 2015; 20: 102-116.
2. Voelkle MC, Oud JHL, Davidov E and Schmidt P. An SEM approach to continuous time modeling of panel data: Relating authoritarianism and anomia. *Psychol Methods* 2012; 17: 176-192.
3. Berry D and Willoughby MT. On the practical interpretability of cross-lagged panel models: Rethinking a developmental workhorse. *Child Dev* 2017; 88: 1186-1206.

4. Masselink M, Van Roekel E, Hankin BL, et al. The longitudinal association between self-esteem and depressive symptoms in adolescents: Separating between-person effects from within-person effects. *Eur J Pers* 2018; 32: 653-671.
5. Kline RB. *Principles and practice of structural equation modeling*. New York, NY: Guilford publications, 2015.
6. Kolenikov S and Bollen KA. Testing negative error variances: Is a Heywood case a symptom of misspecification? *Sociol Methods Res* 2012; 41: 124-167.

**Supplemental material 2: R-code for the RI-CLPM model**

```
RICLPM <- '
### Random intercepts ###
RIucla =~ 1*uclaT1 + 1*uclaT2 + 1*uclaT3 + 1*uclaT4
RIqol =~ 1*qolT1 + 1*qolT2 + 1*qolT3 + 1*qolT4

### within-person residuals ###
Lucla_1 =~ 1*uclaT1
Lucla_2 =~ 1*uclaT2
Lucla_3 =~ 1*uclaT3
Lucla_4 =~ 1*uclaT4

Lqol_1 =~ 1*qolT1
Lqol_2 =~ 1*qolT2
Lqol_3 =~ 1*qolT3
Lqol_4 =~ 1*qolT4

### autoregressive parameters ###
Lucla_4 ~ sx*Lucla_3
Lucla_3 ~ sx*Lucla_2
Lucla_2 ~ sx*Lucla_1

Lqol_4 ~ sy*Lqol_3
Lqol_3 ~ sy*Lqol_2
Lqol_2 ~ sy*Lqol_1

### cross-lagged ###
Lucla_4 ~ cyx*Lqol_3

Lucla_3 ~ cyx*Lqol_2

Lucla_2 ~ cyx*Lqol_1

Lqol_4 ~ cxy*Lucla_3

Lqol_3 ~ cxy*Lucla_2

Lqol_2 ~ cxy*Lucla_1

### within-time associations ###
Lucla_1 ~~ Lqol_1
Lucla_2 ~~ Lqol_2
Lucla_3 ~~ Lqol_3
Lucla_4 ~~ Lqol_4

### some further constraints on the variance structure ###
# The error variances of the observed variables need to be constrained to
zero
uclaT1~~0*uclaT1
uclaT2~~0*uclaT2
uclaT3~~0*uclaT3
uclaT4~~0*uclaT4

qolT1~~0*qolT1
qolT2~~0*qolT2
qolT3~~0*qolT3
qolT4~~0*qolT4
```

```
# The covariance between the intercepts and exogenous variables need to be constrained to zero
# This includes the latent derivatives at T1, which are exogenous variables
Lucla_1~~0*RIucla
Lucla_1~~0*RIqol

Lqol_1~~0*RIucla
Lqol_1~~0*RIqol
'
RICLPM.fit <- sem(RICLPM, data = dataset, missing = "fiml", estimator = "MLR")
summary(RICLPM.fit, standardized = TRUE, fit.measures = TRUE)
```
